# Universal scaling of human flow remain unchanged during the COVID-19 pandemic

**DOI:** 10.1007/s41109-021-00416-0

**Published:** 2021-10-10

**Authors:** Yohei Shida, Hideki Takayasu, Shlomo Havlin, Misako Takayasu

**Affiliations:** 1 Department of Mathematical and Computing Science, School of Computing, Tokyo Institute of Technology, Yokohama, Japan; 2 Sony Computer Science Laboratories, Tokyo, Japan; 3 Institute of Innovative Research, Tokyo Institute of Technology, Yokohama, Japan; 4 Department of Physics, Bar-Ilan University, Ramat-Gan, Israel; 5 Tokyo Tech World Research Hub Initiative (WRHI), Institute of Innovative Research, Tokyo Institute of Technology, Yokohama, Japan

**Keywords:** GPS data, City structure, Power law, Scaling relations

## Abstract

To prevent the spread of the COVID-19 pandemic, governments in various countries have severely restricted the movement of people. The large amount of detailed human location data obtained from mobile phone users is useful for understanding the change of flow patterns of people under the effect of pandemic. In this paper, we observe the synchronized human flow during the COVID-19 pandemic using Global Positioning System data of about 1 million people obtained from mobile phone users. We apply the drainage basin analysis method which we introduced earlier for characterization of macroscopic human flow patterns to observe the effect of the spreading pandemic. Before the pandemic the afternoon basin size distribution has been approximated by an exponential distribution, however, the distribution of Tokyo and Sapporo, which were most affected by the first wave of COVID-19, deviated significantly from the exponential distribution. On the other hand, during the morning rush hour, the scaling law holds universally, i.e., in all cities, even though the number of moving people in the basin has decreased significantly. The fact that these scaling laws, which are closely related to the three-dimensionality structure of the city and the fractal structure of the transportation network, have not changed indicates that the macroscopic human flow features are determined mainly by the means of transport and the basic structure of cities which are invariant of the pandemic.

## Introduction

Since the initial case of infection was confirmed at the end of 2019 https://www.who.int/emergencies/diseases/novel-coronavirus-2019/events-as-they-happen, COVID-19 pandemic has been spreading rapidly all over the world https://covid19.who.int/ and seriously affecting the entire socio-economy such as finance (Goodell 2020; Ashraf 2020), education (Marinoni et al. 2020; Pragholapati 2020) and people’s taste (Ding et al. 2020). As it is well known, human movement play a crucial role in the spread of infection (Tizzoni et al. 2014; Viboud et al. 2006; Balcan et al. 2009; Hufnagel et al. 2004; Colizza et al. 2007; Giles et al. 2020; Gross et al. 2020), and many governments have blockaded cities and restricted individual mobility. Evaluation of human mobility using individuals location data has been studied for Italy during spreading of COVID-19 (Pepe et al. 2020), and there exist many studies, focusing on the changes in human mobility patterns due to the effects of stay-at-home order in each country (Jeffrey et al. 2020; Lutu et al. 2020; Gao et al. 2020; Huang et al. 2020; Yabe et al. 2020; Bonaccorsi et al. 2020; Engle et al. 2020; Jia et al. 2020; Orro et al. 2020). In the United Kingdom, the mobility of people after the Prime Minister’s announcement of an enforced lockdown has decreased significantly (Jeffrey et al. 2020). There has been found a slight difference in the median number of trips between high population density areas and low population density areas, which reflect a decrease in the number of people heading to the city center. In Italy’s national lockdown, the relationship between economic variables and mobility change has been reported, and the results suggest that the lockdown unequally influences the poor (Bonaccorsi et al. 2020). In the United States, the human mobility during the pandemic has decreased significantly on average, but there was a temporarily marked increase in mobility due to the protest demonstration against racism that began with the death of Mr. George Floyd (Huang et al. 2020). In Japan, the number of railway users has decreased significantly, and since the declaration of emergency, the number of station users during peak hours in April and May has declined by 70% compared to normal times https://www.mlit.go.jp/tetudo/tetudo_fr1_000062.html. Despite the non-compulsory measures, the radius of gyration decreased to 50% of the typical and the social contact index reached 30% of normal times by April 15th (Yabe et al. 2020).

In this paper, we analyze human movement patterns during the COVID-19 pandemic from a new viewpoint that focuses on synchronized human flow patterns rather than individual trajectories. Recently, we have developed and applied a new concept of drainage basins analysis in analogous to river flow patterns (Takayasu and Inaoka 1992) to GPS data of 2015 and revealed several universal scaling laws about human flow patterns around big cities (Shida et al. 2020). In contrast to the uncorrelated flow patterns in the afternoon, there are strong flows towards the city center from suburban areas in the morning, and inherent new universal laws of the human flows have been discovered which can be characterized and understood by the 3-dimensional structure of central city buildings and the fractal structure of main transport links. Here, we compare the results of the human flow pattern under the effect of COVID-19 pandemic with the analysis results in the 2015 data (Shida et al. 2020) before the spreading of COVID-19 pandemic.

## Results

### The data

The GPS data was purchased from a Japanese private company https://www.agoop.co.jp/en/. We analyzed the data for all days in 2020. The data consist of user ID, time, latitude, longitude, speed, and direction. The number of users is about 1 million with intervals of data transmission mostly less than 10 min. For protection of privacy, the data transmission stops in the midnight and the user ID is re-newed every day.

### The impact of emergency state issued in Japan

**Fig. 1:**
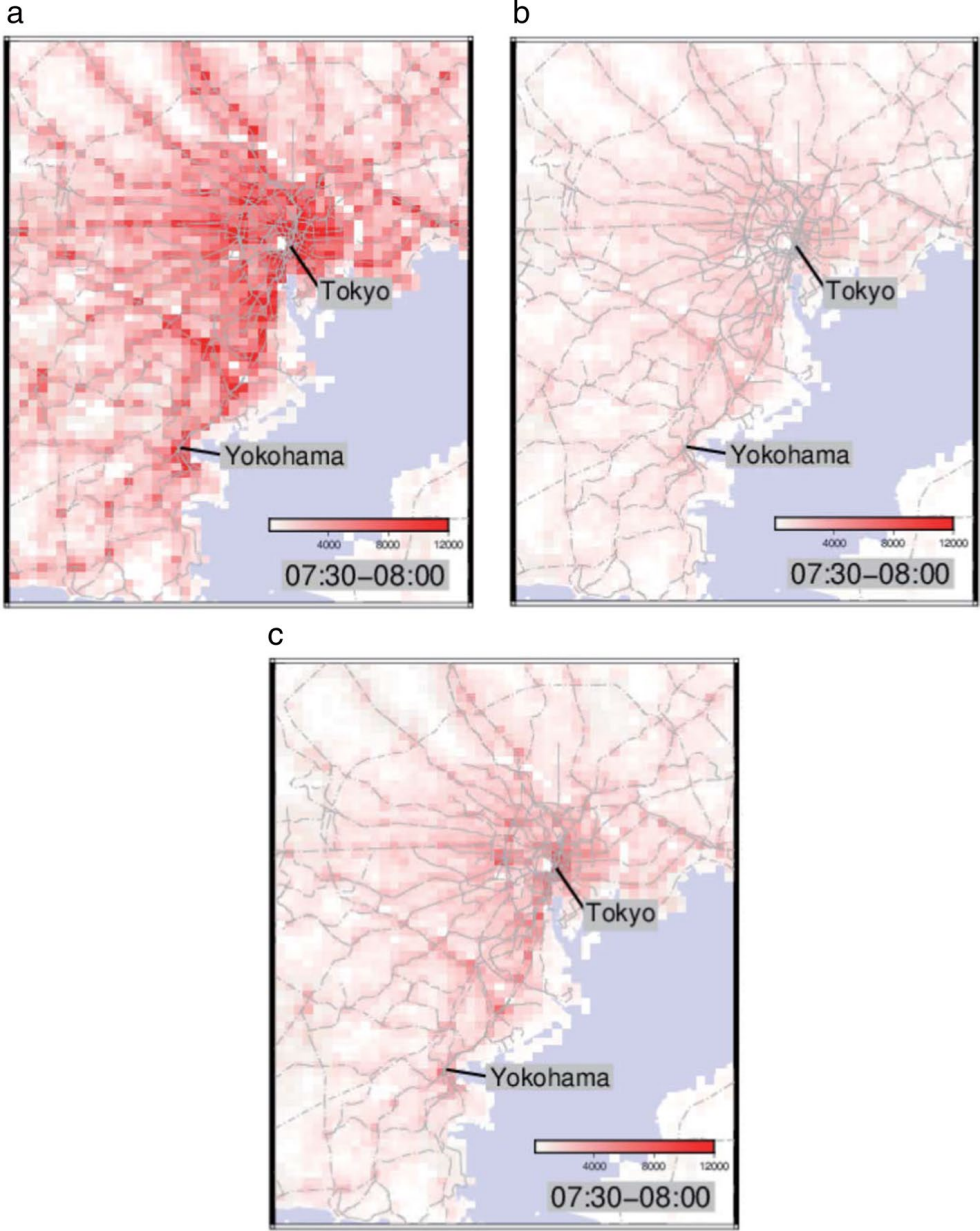
The population distribution of moving people in the morning around the Tokyo metropolitan area. **a**–**c**, Population distribution maps of moving people in the morning on weekdays in January (**a**), May (**b**), and October (**c**), where the predicted population is normalized to the actual population in Japan with color strength proportional to the number of moving people in the square. The total number of moving people in the morning (07:30–08:00) is 9,000,000 (January), 2,500,000 (May), 3,300,000 (October) in the Tokyo metropolitan area, whose population is 35,000,000 (Tokyo, Kanagawa, Saitama, Chiba). The gray lines in the map represent the railways

The declaration of emergency state issued in Japan for COVID-19 from April 7th to May 25th severely restricted the movement of people. In Fig. 1a–c, we show the monthly population distribution on weekdays of moving people in the morning (07:30–08:00) around the Tokyo metropolitan area, in three different months. In contrast to the intense commuter rush in January, we can see that the number of moving people in May has decreased significantly. The population in each square has decreased by about 70% on average, indicating that many people chose telework. In October, the movement of people has revived a little. This is probably because some companies have canceled telework due to reasons such as business productivity and poor internal communication https://www.tokyo-cci.or.jp/page.jsp?id=1023286.

### Drainage basin structures under the effect of COVID-19 pandemic

**Fig. 2:**
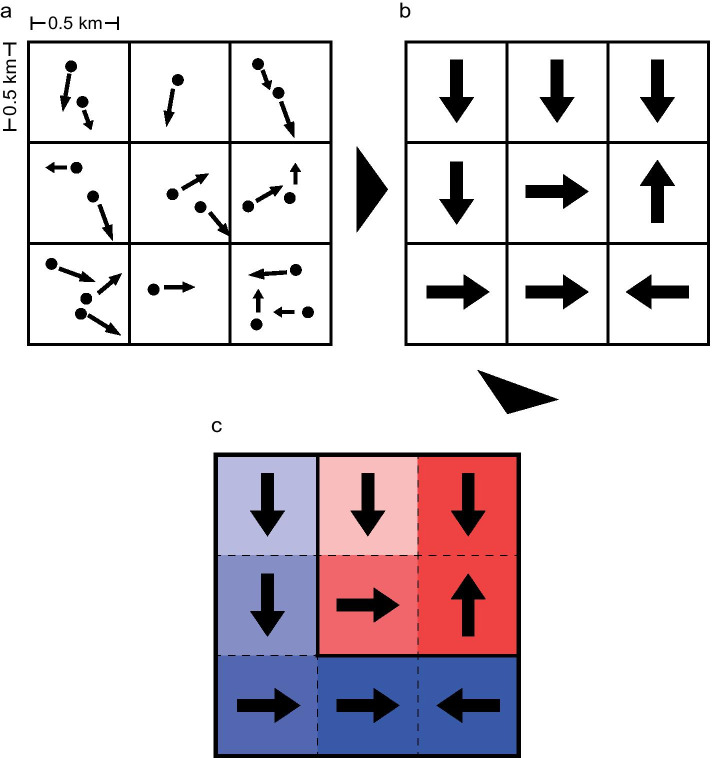
Velocity discretization and The definition of Drainage basins. **a**, The mean velocity in each square of $500 \times 500\,\hbox {m}^2$ is calculated by average velocity over those moving people in the square every 30 min. However, if no one is moving in the square, it is considered missing and not be calculated. **b**, The average velocity of each square is discretized in 4 directions (north, east, south, west) according to the angle. **c**, Each square is connected to an adjacent square according to the direction of the arrow. Connected squares are considered to belong to a drainage basin. There are 2 drainage basins in this figure, red and blue, where the color intensity is proportional to the number of upstream squares

We apply the drainage basin analysis method that has been developed by us Shida et al. (2020) to characterize the human flow patterns during the COVID-19 pandemic, where the analysis range is from April 7 to June 30, 2020 on weekdays, after the state of emergency was announced. Our basin analysis is very simple (Shida et al. 2020). First, in Fig. 2a, b, we divide the map into squares of 500 m $\times$ 500 m square in time intervals of 30 min (5:00–24:00) and calculate the mean velocity vector and the population of moving people in each square. Next, in analogy to river analysis (Shida et al. 2020), we perform the operation of connecting to one of the four adjacent sections according to the direction of the velocity vector of each square. This operation is applied to all squares, and the drainage basin cluster is uniquely determined. Figure 2c shows an example of creating a simple drainage basins. It is possible to divide the focusing area into each basin and further investigate its internal structure. By this operation, it is possible to divide the human flow into several basin clusters, and if there is a flow towards the city center, for example, a huge basin is expected to appear.

**Fig. 3:**
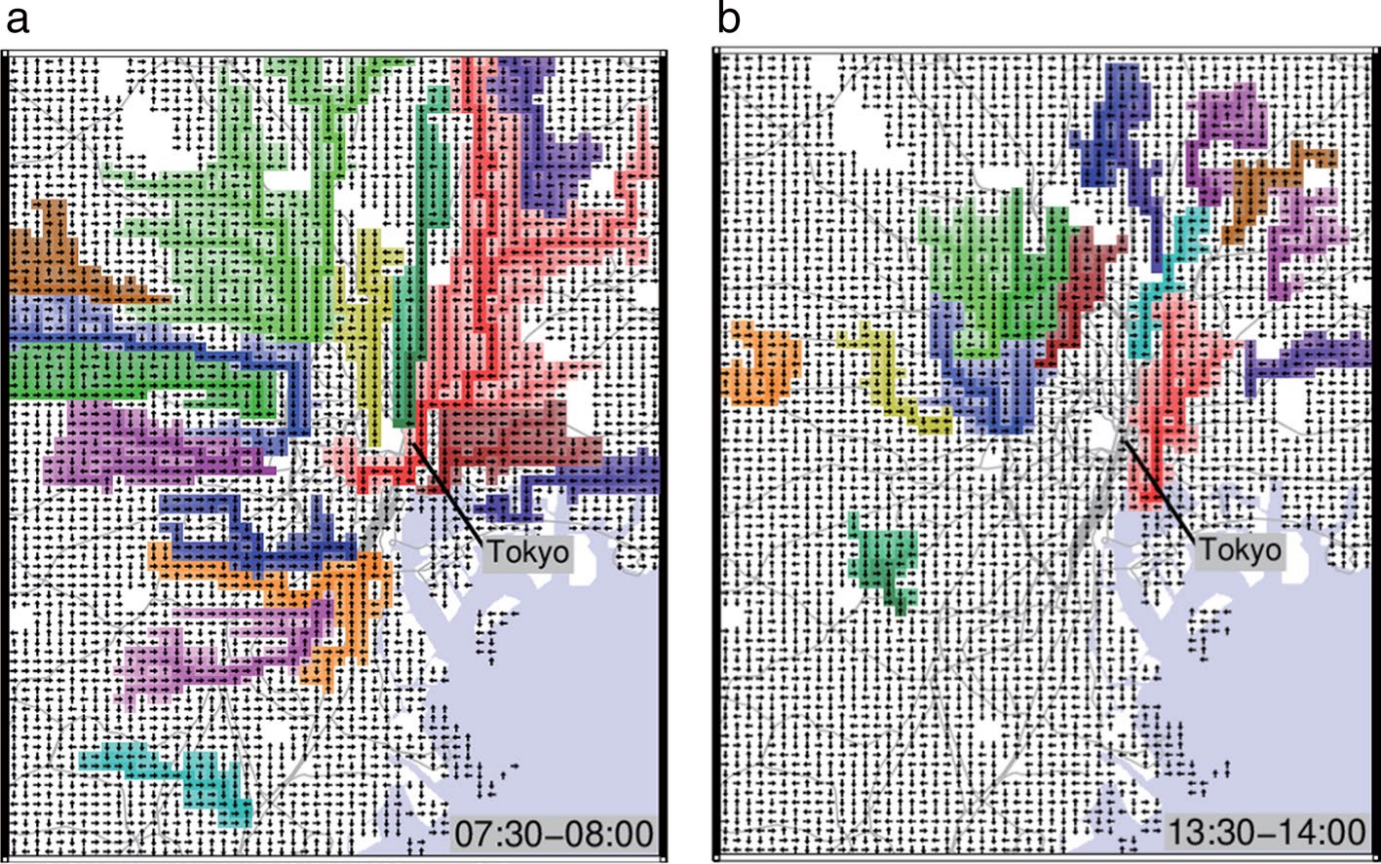
Drainage basins around Tokyo metropolitan area during COVID-19 pandemic. **a**–**b**, Flow maps of basins in **a** the morning commuter rush hour (07:30–08:00) and **b** the afternoon (13:30–14:00) in the Tokyo metropolitan area, where the largest 15 basins are shown in different color codes. The display area is about $20 \times 30$ km. The analysis range is from April 7 to June 30, 2020

Figure 3a, b are top 15 basins drawn in the Tokyo metropolitan area in the morning and noon, where dark squares mean that they are located downstream in the basin. In the morning, we can see that huge basins appear around the center of Tokyo. The basins in the daytime are smaller than the morning basins. At first glance, the pattern of basins look similar to the random patterns observed before the spreading of COVID-19 pandemic, but we can see that the basins are more heading toward the center of Tokyo.

**Fig. 4:**
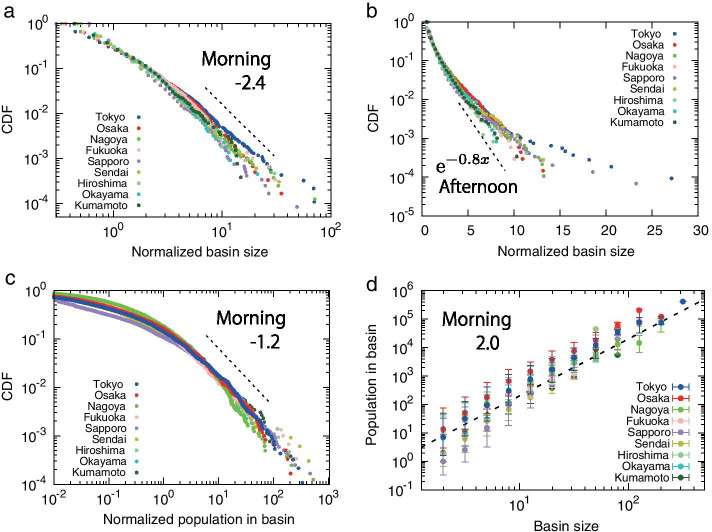
Cumulative distribution function (CDF) of basin size and population distributions for 9 cities during COVID-19 pandemic. **a**, CDFs of basin sizes in the morning rush hour for the 9 analyzed cities (Tokyo, Osaka, Nagoya, Fukuoka, Sapporo, Sendai, Hiroshima, Okayama and Kumamoto) during COVID-19 (from April 7 to June 30, 2020), where basin sizes are normalized by the mean basin size. We prepare human flow that accumulates weekday data from April 7th to June 30th, 2020, and calculate drainage basins. CDFs of the morning commuter rush hour are approximated by a power distribution of the same exponent $-2.4$ as before the spreading of COVID-19 pandemic (Shida et al. 2020). **b**, The afternoon size distribution during COVID-19 pandemic. Comparing with the same plots for the year of 2016 before the pandemic shown in Fig. 8b, the distributions have longer tails deviating clearly for Tokyo and Sapporo. **c**, In the morning rush hours, CDFs of population of moving people in the basin follow a power distribution of exponents $-1.2$, where the population in the basin is normalized by the average population in basins. This is again similar to that found in the non-pandemic times (Shida et al. 2020). **d**, The number of people moving in the basin during the morning rush hours is proportional to the square of the size of the basin

To understand these size differences, we apply the same drainage basin analysis for the nine metropolitan areas of Japan and focus on the size distribution. Figure 4a, b show the cumulative distribution function (CDF) in the morning and noon, respectively. The y-axis shows the cumulative distribution and the x-axis shows the basin size normalized by the mean basin size. The size distributions in the morning are straight lines on the log-log plot and can be approximated by a power law with an exponent value of about 2.4 which are similar to those found in normal times. It can be seen that the basin size distributions of Tokyo and Sapporo in the daytime deviate significantly from a straight line, which is very different from the simulation results (dotted lines). Tokyo and Sapporo took the most amount of time to lift their emergency declarations https://www3.nhk.or.jp/nhkworld/en/news/backstories/1109/, meaning that those cities most affected by COVID-19. The weak directed human flow in Tokyo and Sapporo, which is different from random in the daytime, is probably due to the staggered commuting hours to avoid the infection caused by congestion.

Next, we focus on the number of moving people in the basin during the morning hours. Figure 4c shows that the cumulative distributions of the number of moving people can be approximated by a power law with an exponent value close to 1.2. Figure 1 shows that the number of moving people has decreased significantly due to the COVID-19 pandemic, but surprisingly, the distributions of moving people in each basin have similar power law exponents as before the spreading of COVID-19 pandemic. This suggests that the reduced number of people in each grid is roughly the same fraction of (0.7) each grid. This indicates that the shrink of moving people was uniform, with almost the same fraction. From these power law exponents, the relationship between the size $S_{b}$ and the number of moving people $p_{b}$ in the b-th basin is given as:

1 $$\begin{aligned} p_{b} \propto S_{b}^{2}, \end{aligned}$$

which is invariant although population density decreased. This relationship can be confirmed directly from Fig. 4d.

### Fractal structures

**Fig. 5:**
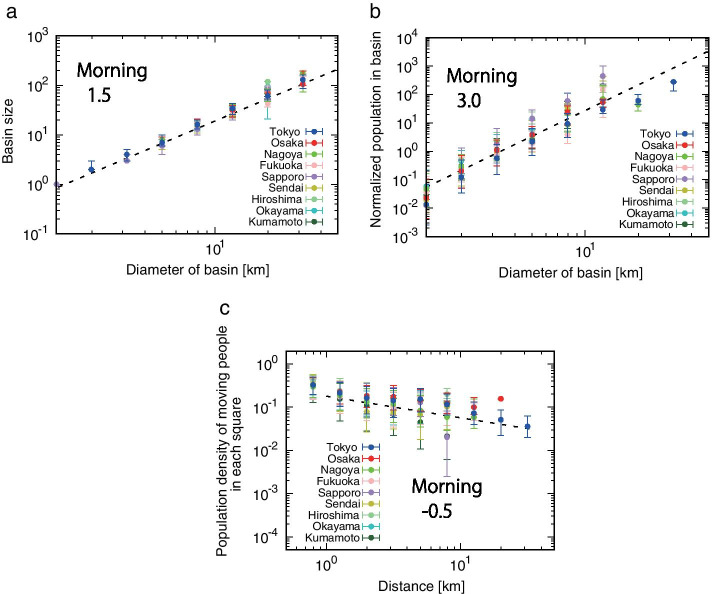
The structure of basins during COVID-19 pandemic. **a**, The size of the basin increases is proportional to the diameter to the 1.5th power. The definition of diameter is the maximum distance between two points in the basin, see reference (Shida et al. 2020). **b**, The number of moving people in the basin is proportional to the cube of the diameter. **c**, Based on the square with the highest density of moving people in the basin, the number of moving people decreases with distance to the power of $-0.5$

Next, we calculate the diameter $L_{b}$ of the basin, *b* , and confirm the universality of the structure of the basin. The diameter $L_{b}$ is defined by the maximum distance between two points in each basin. Figure 5a indicates a size $S_{b}$ is proportional to the diameter $L_{b}$ to the power of 1.5. Using the fractal measure relations (Takayasu 1990), we can estimate the fractal dimension of the basin size. Since the size of the basin is considered to be closely related to the railway and road network, the fractal property does not change despite the significant decrease in the number of people in movement. Furthermore, in Fig.5b, the population of moving people in the basin $p_{b}$ is also proportional to the diameter $L_{b}$ cube, and the three-dimensionality of the city remains maintained. Since it takes many years and labor to change the structure of the city, it is reasonable that the three-dimensional structure of the city did not change during the COVID-19 pandemic. That is, the following relationship

2 $$\begin{aligned} L_{b} \propto p_{b}^{\frac{1}{3}} \propto S_{b}^{\frac{1}{1.5}}, \end{aligned}$$

holds unchanged between the diameter $L_{b}$, size $S_{b}$, and population $p_{b}$ of the basin, despite the significant reduction in the number of moving people to prevent the spread of infection. Finally, in Fig. 5c, we show the relation between population of moving people as a function of distance *r* from the most populated square to reveal the population distribution in each basin. The population of moving people in each square is found to be inversely proportional to the square root of the distance *r*. This is the same exponent as found before the spreading of COVID-19 pandemic, meaning that the government’s declaration of a state of emergency resulted in a uniform voluntary restraint regardless of the region.

## Conclusion

In summary we analyzed the human flow patterns in 9 urban areas in Japan under the COVID-19 pandemic expansion based on the drainage basin analysis of GPS data. Before the pandemic the afternoon basin size distribution was approximated by an exponential distribution, which indicate random movements, however, the distribution of Tokyo and Sapporo deviates significantly from the exponential distribution in the period of government’s declaration of a state of emergency caused by the COVID-19 pandemic. This deviation is probably caused by people’s choice not to travel at peak time in order to avoid congestion. On the other hand, during the morning rush hour, the scaling laws hold universally, even though the number of moving people in the basin has decreased significantly. The fact that these scaling laws, which are closely related to the three-dimensionality of the city and the fractal structure of transportation links, have not changed is considered to indicate that the structure of the city has not changed in spite of movement restrictions conducted. Finally, considering the universality of population distribution within the basin may help prevent congestion. For example, if the arrival time is shifted according to the distance to the destination, the population distribution of moving people in the basin is expected to change significantly. If it is possible to restrict movement for each individual based on GPS data, more efficient infection prevention measures may be possible.

## Figures and Tables

**Fig. 6:**
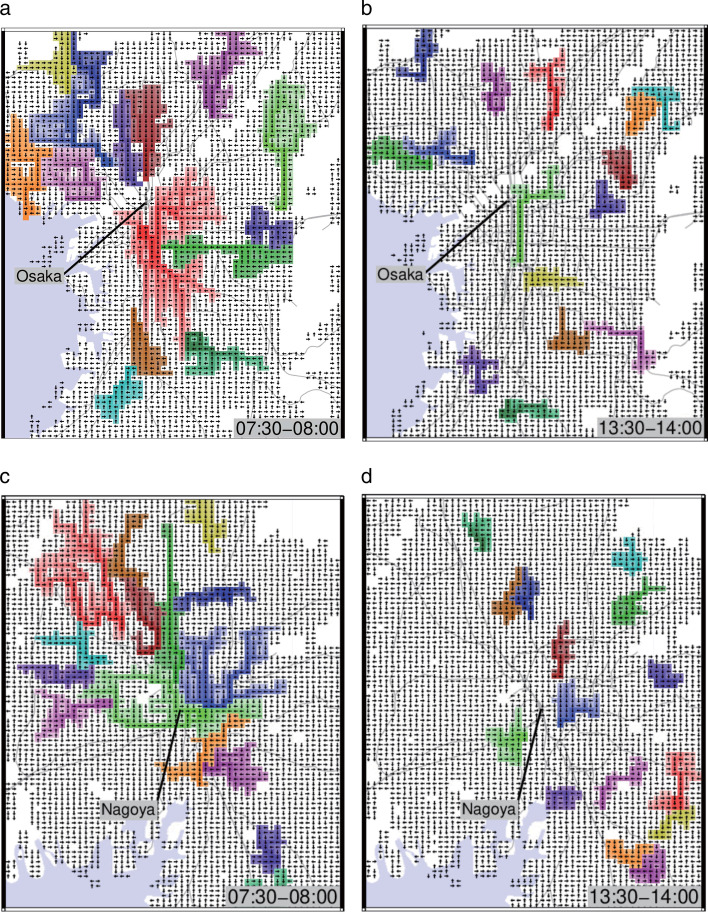
Drainage basins around Osaka and Nagoya metropolitan area during COVID-19 pandemic. **a**, **b**, Flow maps of basins in **a** the morning commuter rush hour (07:30–08:00) and **b** the afternoon (13:30–14:00) in the Osaka metropolitan area, where the largest 15 basins are shown in different color codes. The display area is about 20–30 km square. **c**–**d**, Flow maps of basins in **a** the morning commuter rush hour (07:30–08:00) and **b** the afternoon (13:30–14:00) in the Nagoya metropolitan area, where the largest 15 basins are shown in different color codes. The display area is about 20–30 km square

**Fig. 7:**
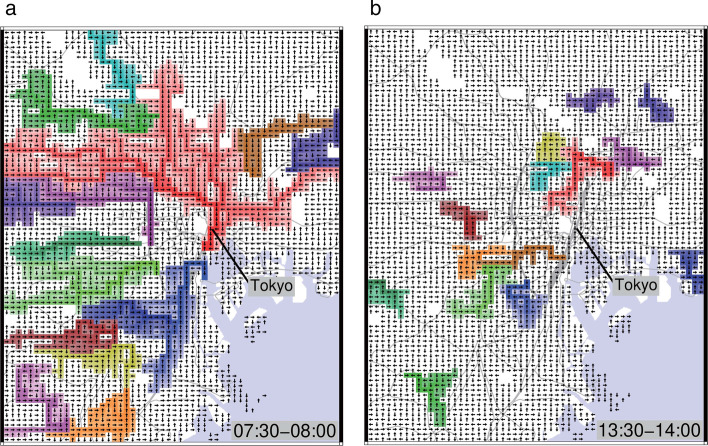
Drainage basins around Tokyo metropolitan area in the year 2016. **a**, **b**, Flow maps of basins in **a** the morning commuter rush hour (07:30–08:00) and **b** the afternoon (13:30–14:00) in the Tokyo metropolitan area, where the largest 15 basins are shown in different color codes. The display area is about 20–30 km square. The analysis range is the whole year of weekday 2016

**Fig. 8:**
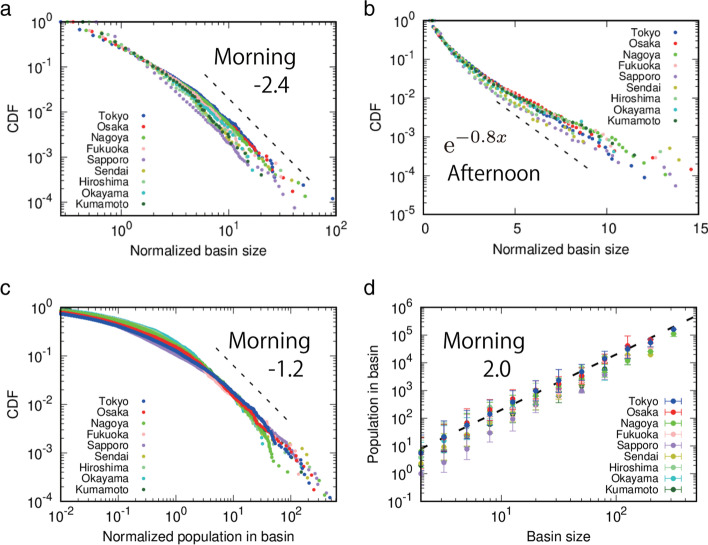
CDF of basin size and population distributions for 9 cities in the year 2016. **a**, Morning and daytime basin size distribution in 9 cities (Tokyo, Osaka, Nagoya, Fukuoka, Sapporo, Sendai, Hiroshima, Okayama and Kumamoto), where the size definition is the number of combined squares. CDFs of the morning commuter rush hour are approximated with a power distribution of the exponent $-2.4$. **b**, The afternoon size distribution can be approximated by the exponential distribution (the dotted line). **c**, In the morning rush hours, CDFs of population of moving people in the basin follow a power distribution of exponents $-1.2$, where the population in the basin is normalized by the average population in basins. **d**, The number of people moving in the basin during the morning rush hours is proportional to the square of the size of the basin. *Note*, (All results are consistent with those shown in Ref. Shida et al. (2020) for the year 2015.)

**Fig. 9:**
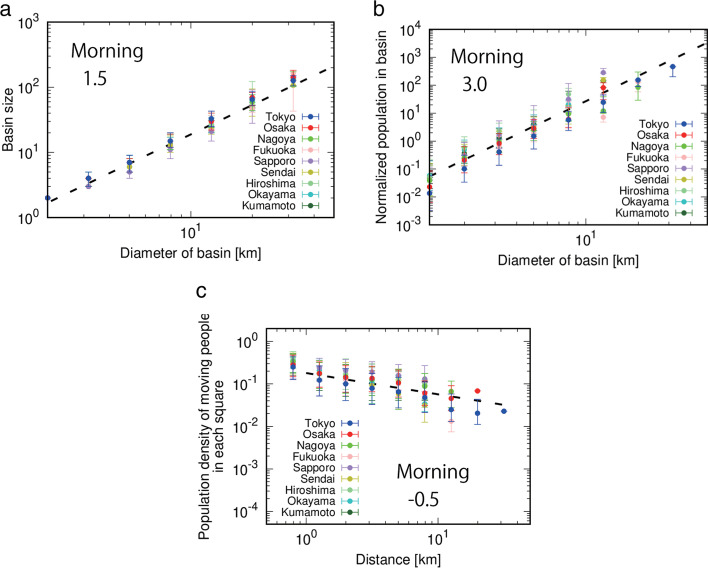
The structure of basins in the year 2016. **a**, The size of the basin increases in proportion to the diameter to the 1.5th power. The definition of diameter is basin crossing, see the paper Shida et al. (2020). **b**, The number of moving people in the basin is proportional to the cube of the diameter. **c**, Based on the square with the highest density of moving people in the basin, the number of moving people decreases to the power of $-0.5$

## Data Availability

Our data cannot be open to public, but the same data can be purchased from a Japanese private company, https://www.agoop.co.jp/en/, which sells “The location information big data which acquired from the smart phone app.”

## Appendix

### Drainage basins around Osaka and Nagoya metropolitan area

Figure 6 show the top 15 basins drawn in the Osaka and Nagoya metropolitan area in the morning and noon.

### Drainage basin structures in the year 2016 before the COVID-19 period

For comparison with Figs. 3, 4 and 5, here we show Figs. 7, 8 and 9 which are produced by the same procedures applied for the whole year 2016 before the COVID-19. All results are consistent with those in Ref. Shida et al. (2020) for the year 2015.

## Abbreviations

GPS: Global positioning system
CDF: Cumulative distribution function
